# Editorial: Understanding the interplay between tumor immune microenvironment and neoantigens for improved immunotherapy

**DOI:** 10.3389/fimmu.2025.1707514

**Published:** 2025-10-15

**Authors:** Sheefa Mirza, Clement Penny, Aijaz Ahmad

**Affiliations:** ^1^ Department of Internal Medicine, School of Clinical Medicine, Faculty of Health Sciences, University of Witwatersrand, Johannesburg, South Africa; ^2^ Division of Pulmonary, Allergy and Critical Care Medicine, Stanford University, Stanford, CA, United States

**Keywords:** neoantigen, tumor immune microenvironment, immune cells, immunotherapy, therapeutic targets

The rise of cancer immunotherapy has transformed oncology, but its success is limited by tumor immune escape, intratumoral heterogeneity, and insufficient neoantigen targeting. Central to immunotherapy’s success are tumor neoantigens - novel peptides arising from tumor-specific mutations - that have the capacity to elicit potent anti-tumor immune responses. However, the tumor immune microenvironment (TIME) frequently imposes complex regulatory networks and immunosuppressive obstacles that limit therapeutic efficacy. Although neoantigen-based approaches offer the promise of personalized and effective treatments, the complex and often suppressive TIME remains a formidable barrier to achieving their full clinical potential. Thus, understanding the complex relationships between TIME and neoantigens is essential for designing more effective, personalized immunotherapies. This Research Topic, “*Understanding the Interplay Between Tumor Immune Microenvironment and Neoantigens for Improved Immunotherapy*,” aggregates cutting-edge studies that elucidate the complex interactions between tumor cells, immune components, and neoantigen landscapes that modulate tumor progression and response to immunotherapy. The collective insights offered by these contributions not only deepen our mechanistic understanding but also pave the way for novel biomarker development and innovative therapeutic strategies.

A central theme across these articles is the understanding that TIME is a dynamic ecosystem shaped by genetic, epigenetic, and environmental signals. The identification and functional characterization of neoantigens provide a crucial entry point into this complexity, as they not only serve as markers of tumor heterogeneity but also act as direct targets for T cell-mediated therapy.

Hu et al. explored the biology, prediction, and therapeutic potential of neoantigens - tumor-specific peptides that arise from somatic mutations - and their role in personalized cancer immunotherapy. The authors described the mechanisms underlying neoantigen generation and T cell recognition via MHC presentation, assessed current methods for neoantigen screening (including DNA/RNA sequencing, mass spectrometry, machine learning-based tools, and molecular docking), and highlighted the limitations, such as false positives, incomplete transcript coverage, and a lack of high-resolution peptide structures. They also outlined potential solutions, such as integrating multi-omics, improving prediction algorithms, and advancing proteomic validation. Finally, they argued that more precise neoantigen identification methods are essential for enhancing the efficacy of vaccines and shaping the future of cancer immunotherapies. 

Complementing this focus on neoantigen biology, Han et al. analyzed over 2,100 publications from 2003 to 2023. The study identified immune cell dynamics - particularly those of CD8+ T cells, regulatory T cells, and tumor-associated macrophages - along with cancer-associated fibroblasts, extracellular matrix remodeling, and immunotherapy as major research hotspots. More recent themes, such as ferroptosis, biomarker discovery, diagnostic signatures, and TME heterogeneity, were also highlighted as growing areas of interest. The authors concluded that advancing these emerging areas, particularly by integrating ferroptosis research with biomarker development and improved characterization of TME complexity, is essential to improving HCC management and therapeutic strategies.

In parallel, the review by Liu et al. offered a detailed overview of the immune cell populations activated by neoantigen-based cancer vaccines, emphasizing how different immune cells interact within the TIME to generate antitumor responses. The authors discussed how transcriptomic profiling and advances in single-cell sequencing have revealed phenotypic signatures in neoantigen-specific T cells, which correlate with vaccine efficacy. The review concluded that improving clinical outcomes depends on characterizing these immune cell types more deeply, optimizing vaccine design and delivery, and strategically combining neoantigen vaccines with therapies that modulate immunosuppression in the TIME.

In another study, Chen et al. reported that extracellular matrix stiffness, abnormal vasculature, and high interstitial pressure hinder TIL infiltration. The article highlighted key molecular pathways that drive these barriers and outlined strategies - such as LOX and YAP/TAZ inhibitors, hyaluronidase treatment, and vascular normalization - to enhance lymphocyte entry and boost the efficacy of neoantigen-based therapies.

The dynamic interplay between therapy and the TIME was further explored by Xu et al., who comprehensively examined the interplay between radiotherapy (RT) and the TIME in pancreatic ductal adenocarcinoma (PDAC). The study highlighted RT’s ability to induce immunogenic cell death, enhance antigen presentation, modulate cytokine profiles, and upregulate immune checkpoint molecules, potentially transforming immunologically “cold” tumors into “hot” ones. However, the dense stroma and immunosuppressive cells in PDAC limit immune activation and therapy effectiveness. The review emphasized the promise of combining RT with immunotherapy and the use of advanced single-cell and spatial transcriptomic technologies to better understand the TME and personalize treatment strategies for improved outcomes in this challenging cancer.

Building on these mechanistic insights, Zhang et al. presented a phase II randomized trial protocol testing the combination of personalized neoantigen peptide vaccines with precision critical lesion radiotherapy (CLERT) in advanced solid tumors. The study aimed to overcome the limitations of neoantigen vaccines in late-stage patients by synergistically enhancing immune activation via targeted radiation to key tumor areas. The study used a 1:1 randomized, open-label, multicenter design, including a crossover option from placebo to vaccine upon progression. The trial assessed progression-free survival and response rates across diverse cancers, leveraging shared neoantigens for broader applicability. This innovative approach integrates precision radiotherapy and neoantigen vaccination, offering a promising platform for advancing combination immunotherapies in challenging advanced cancers.

Together, these studies illustrate how dissecting the interactions between neoantigens and the immune microenvironment can accelerate the development of the next generation of precision immunotherapies. The contributions in this Research Topic highlight the importance of integrating high-resolution mapping of TIME components, advanced computational algorithms for neoantigen prioritization, and rationally designed combination therapies. By linking mechanistic insights with translational strategies, this Research Topic advances our understanding of how tumors evolve under immune pressure and how neoantigen-based interventions can be optimized for clinical benefit.

In conclusion, the contributions gathered here not only expand our knowledge of TIME–neoantigen interactions but also provide a roadmap for developing more effective and durable cancer immunotherapies. We anticipate that these insights will inspire continued interdisciplinary research, ultimately enhancing the precision and efficacy of immunotherapeutic strategies for cancer patients worldwide.

